# Development of a Binational Framework for Active and Healthy Ageing (AHA) Bridging Austria and Slovenia in a Thermal Spa Region

**DOI:** 10.3390/ijerph18020639

**Published:** 2021-01-13

**Authors:** Sonja Lindner, Kai Illing, Josef Sommer, Tatjana Krajnc-Nikolić, Johann Harer, Christoph Kurre, Karl Lautner, Mateja Hauser, Daniel Grabar, Robert Graf-Stelzl, Christian Korn, Klaus Pilz, Bernhard Ritter, Regina Roller-Wirnsberger

**Affiliations:** 1 Department of Internal Medicine, Medical University of Graz, 8036 Graz, Austria; sonja.lindner@medunigraz.at; 2 Faculty of Health Management in Tourism, University of Applied Sciences FH JOANNEUM, 8344 Bad Gleichenberg, Austria; kai.illing@fh-joanneum.at; 3 Austrian Thermal Spas and Health Resorts Association, 1140 Vienna, Austria; j.sommer@kaiservon.at; 4 RU Murska Sobota, National Institute of Public Health, 9000 Murska Sobota, Slovenia; tatjana.krajnc-nikolic@nijz.si; 5 Human Technology Styria GmbH, 8010 Graz, Austria; johann.harer@human.technology.at (J.H.); christoph.kurre@hotmail.com (C.K.); 6 ÖVP Bad Radkersburg, Municipality of Bad Radkersburg, 8490 Bad Radkersburg, Austria; karl.lautner@gmx.at; 7 Directorate of Dosor Home for the Elderly, Tečni&Srečni, 9252 Radenci, Slovenia; mateja@tecnisrecni.com; 8 General Hospital Murska Sobota, 9000 Murska Sobota, Slovenia; daniel.grabar@sb-ms.si; 9 Styrian Hospitals Limited Liability Company KAGes, 8160 Federal Hospital Wei, Austria; robert.graf-stelzl@kages.at; 10 Parktherme Bad Radkersburg Thermal Spa, 8490 Bad Radkersbur, Austria; christian.korn@badradkersburg.at; 11 Radkersburger Hof Center for Rehabilitation, 8490 Bad Radkersburg, Austria; klaus.pilz@radkersburgerhof.at; 12 Department of Internal Medicine, Federal Hospital Wagna, 8435 Wagna, Austria; bernhard.ritter@kages.at; 13 Department of Internal Medicine, Federal Hospital Bad Radkersburg, 8490 Bad Radkersburg, Austria

**Keywords:** active and healthy ageing, cross-border collaboration, community participation, healthy ageing region

## Abstract

In view of ongoing demographic developments resulting in a longer life expectancy of the European population, the creation of “age-friendly” environments represents an initiative picked up by the European Union and its Member States to enable active and healthy ageing. The present study aims at the co-creation of a cross-border framework model to deploy a healthy ageing region linking Austria and Slovenia, building on previous work dealing with the development of an integrated regional ecosystem for active and healthy ageing. A qualitative, community-based action research method based on focus group discussions allowed the development of an exemplary framework model for active and healthy ageing building on cross-border collaboration in the region of Promura. Within the project group, twelve cross-border regional key assets were identified. In the course of further open discussions, an exemplary model for the deployment of a cross-border healthy ageing region was developed, comprising underlying fundamental environmental aspects, regional structures in the field of health and care as well as crosscutting features spreading across all levels. This article presents a promising, strategic co-creation approach on how to span a model on active and healthy ageing across two cross-border regions with similar characteristics and assets.

## 1. Introduction

The European population is enjoying longer lives [1]. Still, differences in health and social care systems as well as years spent in good health and wellbeing, however, exist among European Union member states (EU-MSs) [2]. In this context, Austria has a strong focus on institutional health and social care. Slovenia, in contrast, has been focusing on the development of care services nested in primary health and social care, setting up one of Europe’s first primary health care centers [3]. As a matter of fact, Austria ranks higher than Slovenia in self-reported population health in OECD (Organization for Economic Co-Operation and Development) data for health and wellbeing during the ageing process [2]. Both countries, being partners in Europe, have a long-standing tradition in joint and cross-border activities—also within specific funding schemes—with continuous communication from micro- to meso-and macrolevel in different political systems, from transport to education to health and social care [4,5]. 

A population living longer, healthy, and independent remains an aspiration targeted by the EU and is supported by its MSs, also through participation in the European Innovation Partnership on Active and Healthy Ageing (EIPonAHA). EIPonAHA is an action initiated by the European Commission to stimulate innovation, research, and digital transformation in the context of active and healthy ageing [6]. One of the major pillars for joint developments across regions in Europe under this umbrella is the creation of “Age-friendly environments”, with the aim to the design, establish, and co-create environments or ecosystems that enable European citizens to age actively and healthy [7]. Despite these commitments towards healthy ageing, several challenges persist, requiring effective responses to an ageing population. Especially the ageing process is highly heterogeneous with no universal representation of an older person. Moreover, the environment where we live in and interact with each other is likely to induce diversity in older age, often leading to health inequities. Ageism and changes in technological developments or globalization amongst others remain further implications emphasizing the need for comprehensive and flexible healthy ageing responses [8]. Therefore, the World Health Organization (WHO) claimed the development of age-friendly environments as a strategic objective in its “Global strategy and action plan on ageing and health” [9]. The creation of such age-and ageing-friendly environments covers a variety of sectors, such as transports, housing or health and social care and requires activities and commitment of various actors [9]. Sixsmith et al. [10] highlighted the importance of interaction of communities, service providers and older people themselves in order to establish an environment, where ageing well in place is enabled by building sustainable partnerships. Especially frail older people claim an increased need of adapted environmental characteristics to support ageing in place [11].

Very recently, an evidence-based methodology to create ecosystems for active and healthy ageing has been published [12]. Although this model provides guidance and a rationale for engaging different stakeholders in active and healthy ageing across the life-course activities, a structured and integrated development of healthy ageing regions remains highly context-sensitive. Based on this previous work and the aim of the EU Commission to connect European regions based upon their capacity profile [13], the current publication describes how to make use of community-based action research to deploy active and healthy ageing in a cross-border bridging between Austria and Slovenia in the region of “Promura” (Austria—region Bad Radkersburg and north-eastern Slovenia—region Murska Sobota). Both areas share a similar population structure with 29.5% of the population in Bad Radkersburg and 25.8% of the population in Murska Sobota being older than 65 years [14,15], indicating the priority of age-appropriate products and services to enable social participation and independence in older age. As an outstanding feature, the cross-border area introduced offers an extensive thermal infrastructure with curative spring water and specific health promoting activities. Six thermal spring locations in southeastern Styria and two thermal spring locations in the adjacent Slovenian region provide a range of resources and offers in the field of wellness, health and regeneration, benefitting both the regional population as well as tourists [16,17]. The need to put more strategic focus on targeted regional development and foster implementation of cross-border collaboration and activities in these close-border areas has been determined in the Styrian Economic and Tourism Strategy 2025 recently [18]. Regional capacities with a high density of health prevention offers in primary care and a high density of health professionals and health tourism institutions seem a perfect model for joint bundling and complementary cross-border utilization of resources towards a shared ecosystem for active and healthy ageing. Therefore, the aim of this publication was to outline a strategic framework model that promotes linkage of pre-existing competences across the Slovenian–Austrian border and encourages regional development and innovation in the field of active and healthy ageing.

## 2. Materials and Methods 

### 2.1. Design

In order to pursue framework model development within an integrated, cross-border active and healthy ageing (AHA) ecosystem in a particular thermal spa region, a community-based action research approach was chosen. There are several rationales supporting the method of community-based research: it connects partners with diverse skills and expertise to address a complex problem; it has the potential to bridge the partners included and it enables to create a theory that is grounded in social experience and leads to more effective practices [19]. As trust, appreciation of cultural differences, respect, open communication, and research commitment were recently identified as cornerstones of community-based participatory research [20], focus was put on these elements in order to facilitate a successful interplay of science and practice. This co-creation approach allows an in-depth exploration of complex social issues or underlying patterns of behaviour in a specific setting, also revealing the meaning of these patterns for stakeholders in a particular context. The study participants collaboratively address a thematic concern and elaborate a deeper understanding or improvement of local situations or practices that are empowering them to undertake further strategic actions and interventions [21,22]. 

### 2.2. Sampling

Participants from Austria and Slovenia were selected with the pursuit to include regional experts that are able to contribute with relevant information and offer valuable insights to the needs, issues, and concerns of their respective communities. All participants gave their informed consent to participate in the study beforehand. Experts’ participation is justified upon their professional involvement, by representing major regional organizations and institutions in the field of health and/or tourism and/or active and healthy ageing and policy, ensuring that all possible fields and dimensions of the topic under consideration were covered. As all eligible experts are well settled in the cross-border region and already interact with each other on a regular basis, sample selection was easily facilitated. Regional experts meeting these criteria were invited to a local introductory meeting where the three academic experts (KI, RRW, and SL) presented the initiative. All eligible experts initially invited attended the meeting and gave their approval for participation. During the project and within this publication participants are summarized as “Promura project group”. The Promura project group focuses on the promotion and facilitation of cross-border cooperation in the fields of tourism, health and care with the overall aim to foster competitiveness and develop an integrated cross-border strategy for active and healthy ageing.

### 2.3. Data Collection and Analysis

A total of four action research focus groups was conducted from November 2018 until February 2019, following a multi-stage approach. The focus group method was chosen in order to create a highly interactive environment and enable participants to immediately respond to each other’s ideas and opinions, therefore considering as many aspects as possible and maximizing the data generated. Data retrieved from the preceding focus group meeting were used in the following focus group session in order to adjust the qualitative methodology to outcome and strategy development. A co-creation approach was applied with one expert of the University of Applied Sciences Graz (KI) being responsible for design and organization of the focus groups and two experts of the Medical University of Graz (RRW, SL) supporting focus group management alongside. 

The first focus group session dealt with the discovery of regional fields of strength in the context of healthy ageing and health tourism. Key topics were elaborated by applying a content analysis following an example of health regions in Germany [23], condensed and quantified until saturation was reached and additional data did not reveal any new information. Based on the previous outcome, in the second focus group session a qualitative benefit analysis was carried out in order to emphasize and defend the quantitative results. The different strategic characteristics elaborated during the first workshop were rated with particular reference to the priority of following aspects: (1) Preservation and expansion of existing business; (2) Settlement of future-oriented economy and job creation; (3) Quality of life and cultural life; (4) Suitability of existing resources. Participants of the second workshop were additionally asked to rate the importance of each key topic from 0 to 9. The two ratings for each item were multiplied and allowed an overall ranking position of each key topic [24].

**Figure 1:**
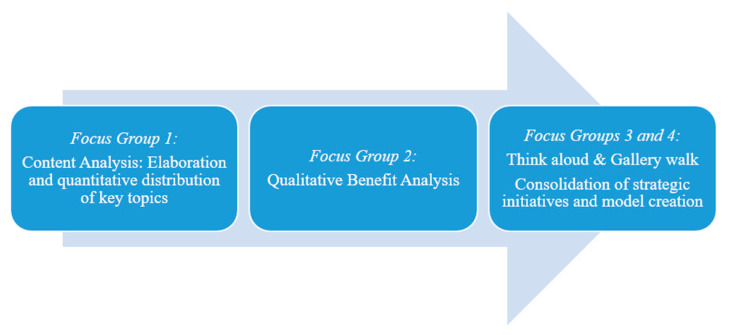
Illustrates the methodological approach applied for the consolidation and development of a cross-border model for active and healthy ageing.

To condense this content prioritized in the first two workshops, the third meeting was run using the think aloud method [25]. The key topics arising from the previous work were analyzed during workshop three on the basis of their coverage area on the micro-, meso- and macrolevel and upon their potential to foster regional capacity building. The third workshop was run in open discussion among participants until saturation was reached and no new findings emerged. This third focus group meeting was transcribed according to the method of Mayring [26] to facilitate data analysis, using an open-coding process of transcripts. Codes and categories selected for analysis were: conceptualization, funding and cross border collaboration options with special focus on accumulative data, conformities and similarities of discussion feedbacks from participants. In the fourth and last focus group session we used think aloud method combined with the format of “gallery walks” with the aim to condense the content and align strategic procedures for implementation of the cross-border framework with exemplary character [27]. This final process of prioritization and concretization fed into strategic focal action points that build a regional strategic model for active and healthy ageing. Figure 1 demonstrates the step-based mixed-method approach described in this section.

Figure 1: Methodological approach for developing a cross-border framework model for active and healthy ageing.

### 2.4. Ensuring Rigor

In qualitative research, rigor may also be understood as “trustworthiness” by meeting criteria of credibility, transferability, dependability, and confirmability [28]. In the research presented in this publication, distinct verification strategies were utilized in order to ensure validity and reliability, and thus, rigor. These strategies are integrated in each step of the project development process in order to identify inaccuracies and establish a self-correcting mechanism [29]. Within this research project, sample appropriateness was guaranteed by involving participants with proven expert knowledge in the research topic and by striving for generating sufficient data to cover all aspects of the topic under study. Moreover, data collection and analysis were performed timely and simultaneously, following an approach of iterative interaction. Not only did we include definite data collection, but tried to include non-verbal expressions, the possibility to react sensitively to focus group participants and to ensure reliability among researchers (all included as Promura project group). 

Researchers in the group aimed at constantly shifting content analysis between the micro-level of data and the macro-level of the overall underlying theoretical concept of a cross border active and healthy ageing ecosystem, following the concept of theory development. In qualitative research, the investigator is part of the topic under study and will inevitable influence it. Researchers’ creativity, sensitivity, flexibility, and responsiveness is another conditional aspect for meeting research validity and reliability [29]. This is why a dual research experts’ assistance as described in Section 2.3 was chosen to facilitated a high degree of observation of non-verbal expressions, the possibility to react sensitively to focus group participants and to ensure reliability among researchers [30]. 

Following this research approach it was finally possible to develop an overall framework model of a cross border ecosystem for active and healthy ageing for the region of Promura.

## 3. Results

### 3.1. First Stage Results—Strategic Key Assets

The Promura project group comprised of experts and regional stakeholders in the field of tourism, wellness, health and social care, politics, and education from Austria and Slovenia. Three experts from Slovenia and eleven experts from Austria participated in the process, covering the following areas of expertise within regional key institutions and organizations: directorate of the regional hospital (SLO), directorate of the regional long-term care nursing home (SLO), lead of the regional unit of the national institute of public health Slovenia (SLO), head of the regional tourism association (AUT), human technology (AUT), medical directorate of regional health resorts and clinics (AUT), directorate of regional hospitals and long-term care centers (AUT), directorate of regional school of nursing (AUT), mayor’s office and local government (AUT), head of the national spa and health resorts association (AUT), directorate of the regional thermal bath (AUT), and directorate of regional rehabilitation and health center (AUT). Each focus group was held for 2 h with the 14 expert participants and guided by three researchers (see Section 2.3). In the content analysis of the first focus group, 20 key topics in the field of active and healthy ageing representing strengths of the cross-border region Promura were elaborated. An open discussion round at the end of the first workshop facilitated comprehension of content to principle 12 strengths and opportunities as strategic priorities for the cross-border region. In the second workshop and focus group session, the qualitative benefit analysis emphasized the outcome of the previous session with only minor shifts in prioritization. As can be seen in the numbered list below, Ageing, Education and Training, Networking/Organization/Business development, Medical Care, Living arrangements/Housing development, and Health tourism evolved as leading strategic focal point areas followed by the topics Prevention and Health promotion, Nature/Experience of Nature, Outdoor-sports, Nutrition, Mobility, and Complementary Medicine. In the following open discussion, all participants agreed with the result and gave consent to proceed within these topics with greater in-depth focus groups.

Cross-border regional strengths and opportunities detected during the first workshop:

1. Ageing
2. Education and Training
3. Networking/Organization/Business development
4. Medical Care
5. Living arrangements/Housing development
6. Health tourism
7. Prevention and Health promotion
8. Nature/Experience of nature
9. Outdoor-sports
10. Food and Nutrition
11. Mobility
12. Complementary Medicine

### 3.2. Second Stage Results—Developing a Framework Model for the Cross-Border Region Promura

**Figure 2:**
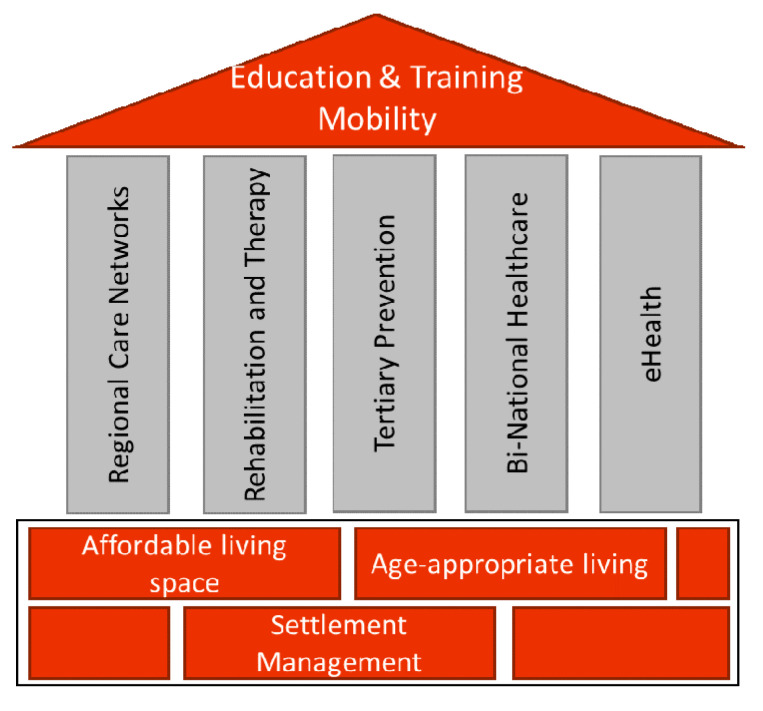
Shows the exemplary framework model elaborated within the community-based action research approach for strategic deployment of a cross-border ecosystem for active and healthy ageing. The regional environment with living and settlements aspects builds the contextual foundation. Regional care networks, rehabilitation and therapy, prevention, cross-border healthcare and e-health are pillars illustrating regional structures and capacities to enable active and healthy ageing. The cross-cutting issues education, training and mobility build an overarching feature permeating the regional structures on all levels.

Data stemming from the third and fourth workshop focus-group sessions comprised strategic core ideas covering the regional micro-, meso-, and macrolevel in alignment with the previously identified key topics by applying the think aloud method [25]. Within an informal open coding process, 27 general categories could be built, all of them relating to the “branding” of regional capacity building. The further prioritization and concretization process led to 10 strategic objectives and cornerstones. Within an open discussion process, these objectives were schematically aligned towards their structural characteristics. Living and settlement aspects are regarded as environmental elements that build the foundation for a cross-border healthy ageing region. Regional health and care structures that build on existing capacities and are aimed towards stronger strategic positioning in this field form the core essence in this context. Education and training as well as mobility constitute an overarching thread that needs to be integrated on all levels. Figure 2 shows the final exemplary model for deployment of a cross-border region for active and healthy ageing.

Figure 2: Exemplary model for deployment of a cross-border region for active and healthy ageing

These results represent a bouquet of different focus topics, clearly addressing the fields health and care and emphasizing existing regional competences. By further analyzing the results it became obvious that individual results could be linked with each other, meeting overarching needs and requirements of the region by bundling joint efforts, reinforcing the intention to create a binational framework for active and healthy ageing.

## 4. Discussion

The EU Biodiversity Strategy to 2020 calls on Member States to carry out a mapping and assessment of ecosystems and their services [31]. This terrestrial ecosystem of the region Promura between Austria and Slovenia shares communalities in water, hot springs, and biospheres. People living in the region are either Austrian or Slovenian citizens. People settled in the region of Promura share epidemiological and biological similarities independent from citizenship. This means that the population in the cross-border region is ageing with a percentage of 29.5% of the population in Austria, city of Bad Radkersburg, and 25.8% of the population in Slovenian part of Promura in the city of Murska Sobota being older than 65 years [14,15]. This rapid shift in population characteristics forced politically responsible stakeholders to rethink the regional capacities raising the shared wish for a cross-border regional development to satisfy the needs of an ageing population making use of region strengths and capacities to support healthy ageing.

One challenging aspect in this regard is the fact that a consensual definition of the term “healthy ageing” is lacking, although definitions and common features are thoroughly described in scientific literature [32]. However, as older people constitute a heterogeneous group with diverging ageing processes, a uniform and restrictive understanding of healthy ageing may not be helpful for developing age-friendly environments and therefore underpins the necessity of context-sensitivity [33]. 

The current publication describes a process of co-creation towards a cross border- ecosystem for active and healthy ageing making use of academic potential to drive regional, structural developments within the light environmental communalities between the two countries [34]. Researchers involved in the cross-border ecosystem development made use of qualitative research methods to bring together Austrian and Slovenian regional stakeholders and to capitalize existing capacities in a highly participative approach. 

Basically, the concept of active and healthy ageing is anything but new for the region, considering that recently a Styrian evidence-based ecosystem for active and healthy ageing was developed, pointing out strong regional capacities in the field concerned [12]. Borrmann et al. demonstrated the need to connect regional stakeholders and develop synergistic initiatives that help to pursue a common strategy for active and healthy ageing. As a prerequisite, end-user of healthy ageing products and services as well as stakeholders and professionals call for transparent communication and information transfer about processes in this field that directly concern them [12]. This current publication may be regarded as continuation of the process presented by Borrmann et al., since it picks up the status quo of the Styrian model, tailored towards regional fields of strength within a community-based co-creation process and strategically positions the cross-border region in the field of active and healthy ageing, engaging in the increase of its competitiveness.

As may be seen from Figure 2 the regional capacities elaborated during the process are strongly aligned with the environmental ecosystem present in the cross-border region of Promura. This confirms the strategy of the EU commission to strengthen regions and territories independent of national MSs and aligned with the regional biodiversity strategy [35]. Promura shares cross-border capacities in water, hot springs, and biospheres giving rise to strong health tourism offers, also including rehabilitation and tertiary care prevention for visitors and guests (Figure 2). During the project participants reflected, how to best make use of the market offers in place in Promura for health tourism and open the capacities for tertiary prevention for the regional ageing community. Especially the benefit of tourism on active and healthy ageing remains a widely discussed topic in scientific literature [36,37], also with regards to the advantageous effect of healthy water-based tourism [38] as it is offered in the Promura region.

Given the current pandemic with SARS-CoV-2 virus the European Commission presented an approach on how to restore mobility and tourism’s recovery once the situation allows it. One aspect mentioned in this publication refers to the connection of local and regional citizens to local tourism offers, narrowing the perspective towards a more regional level [39]. Not only will local resources and traditions be promoted, but also the values of a territory, will be appreciated, helping to preserve heritage and complement existing economic actions [40]. The World Tourism Organization [40] also recommends that national, regional, and local governments should create an enabling environment, helping to realize the potential of tourism to promote investments, innovation, digitalization, and skills, amongst others. The approach described in this current publication will help to restore confidence, to stimulate investment, innovation, education and training and as such will promote the development of high-quality health tourism in the context of active and healthy ageing.

Cross-border mobility and binational healthcare and education for professionals were regarded as appealing albeit complex targets worth striving for during the creation of a cross-border ecosystem for AHA. In general, cross-border healthcare is embodied in law in Article 168 of the Treaty on the Functioning of the European Union (TFEU), encouraging cooperation between Member States in cross-border areas [41]. The intention to collaborate in healthcare provision across borders is likely to arise between regions that share similar social welfare, are geographically close to each other or share a common history [42]. Although these aspects widely apply to the region presented in this publication, there are crucial factors to consider, all bearing the potential to facilitate or hinder cross-border cooperation in healthcare. These factors can be classified into four dimensions: Geographical/demographic aspects, economic/technological aspects, cultural/societal aspects, and regulatory aspects [42]. Especially the regulatory dimension was thoroughly discussed in the focus group sessions. Not only do general legal factors influence the extent and possibility of cross-border collaboration, but healthcare system factors such as financing, remuneration, characteristics of each domestic health care system and health care services constitute critical issues to solve [42]. Demand on cross-border processes is high, as they need to overcome discontinuities resulting from the differences mentioned [43]. Regional cooperation as discussed bears the potential to drive knowledge sharing, joint training of health professionals and development of specialized healthcare units in border regions; nevertheless, it may not achieve the full potential that is understood by the concept of cross-border healthcare cooperation [42].

Given the current challenging situation posed by the ongoing Covid-19 pandemic with travel restrictions gravely hitting the tourism sector, innovation and transformation is now more important than ever. This current de-globalization process, although bringing with it devastating impacts never thought possible, may open the door to newly shaped paths in (health) tourism and leisure [44]. Despite the fact that this publication does not provide overall coping strategies for the current Covid-19 crisis, it illustrates an example on how to launch a strategic process that helps to identify regional strengths in health tourism and shape the strategic identity in an ever-changing context. 

Our work contains strengths and limitations. As an additional experience of the process and central factor for success, new cross-sectoral relationships were established and sustainably maintained, facilitating regional anchoring of the elaborated framework for active and healthy ageing. By applying the community-based action research method, already existing strengths and resources within the Promura community were utilized and collaborative partnerships were deepened. These aspects were also thoroughly discussed in literature and underpin the adequacy of the method used for the topic under study [19]. The geographic accessibility of the cross-border area, the socio-cultural proximity, similar institutional context conditions and an already established cross-border integration represent ideal framework conditions for cross-border actions [45]. Moreover, the community-based co-creation process enabled the research team to gain insight into regional capacities, cross-border initiatives, as well as attitudes and knowledge regarding health tourism and healthy ageing. The high degree of citizen and stakeholder participation across different sectors facilitated representation and identification of participants with the strategic goals elaborated. This publication picks up on the development process of the Styrian ecosystem for healthy ageing [12] and reinforced taking the next step forward towards strategy implementation.

Major limitation of the work presented is the ratio between representatives from Austria and Slovenia, which was not yet distributed equally with more Austrian stakeholders participating in the work group. This imbalance may be justified by language barriers and may result in an under-representation of local interests and concerns of Slovenian stakeholders. As Foley and Timonen [46] pointed out, quality of data generated within focus groups is highly dependent on the group composition. Moreover, the conduct of a community-based research based on cross-border collaboration presumes interaction in a highly social context with complex relationships where distinctions between science and practice as well as between individual backgrounds of the stakeholders involved may blur. Successful collaboration highly depends on the willingness of persons concerned to participate in open discussions, be willing to adopt different viewpoints and accept vulnerability to a certain extent. A challenge worth mentioning in this specific regard is that the persons involved all represented a particular position and perspective and may focused on interests that primarily correspond with their perspective but as a result may diverge within the whole project group. Gaps regarding power relations of the parties concerned also may remain an obstacle in any collaboration effort. The development of ethical guidelines and principles for community-based research may help to overcome the challenges addressed [47]. As the study concentrated on a cross-border region between Austria and Slovenia, a truly regional perspective was taken. This may inhibit the possibility for translation and adaptation of the approach for following regions. In addition, the study conducted represents pioneer work of cross-border collaboration in the Promura region with no preceding experience values or data available. Further research in this area, for example data presentation by case studies, is recommended. Nevertheless, the work presented underlines the importance of tailoring strategic development approaches towards healthy ageing to the local and regional context.

## 5. Conclusions

In the light of demographic developments, cross-border collaboration in the field of health and care represents a distinguished tool to utilize available regional capacities and resources and to bundle existing expertise towards active and healthy ageing. The elaborated model illustrated in this publication is based on a co-creation process and integrates already present resources with strategic objectives and cross-cutting issues between two cross-border regions with similar characteristics and high-level competence in health, care and health tourism.

## Data Availability

All relevant data are contained within the article.
